# Connection Lost, MAM: Errors in ER–Mitochondria Connections in Neurodegenerative Diseases

**DOI:** 10.3390/brainsci11111437

**Published:** 2021-10-28

**Authors:** Ashu Johri, Abhishek Chandra

**Affiliations:** Feil Family Brain and Mind Research Institute, Weill Cornell Medicine, New York, NY 10065, USA; abchandra2010@gmail.com

**Keywords:** mitochondria associated membranes (MAMs), Alzheimer’s disease, Huntington’s disease, Parkinson’s disease, amyotrophic lateral sclerosis

## Abstract

Mitochondria associated membranes (MAMs), as the name suggests, are the membranes that physically and biochemically connect mitochondria with endoplasmic reticulum. MAMs not only structurally but also functionally connect these two important organelles within the cell which were previously thought to exist independently. There are multiple points of communication between ER–mitochondria and MAMs play an important role in both ER and mitochondria functions such as Ca^2+^ homeostasis, proteostasis, mitochondrial bioenergetics, movement, and mitophagy. The number of disease-related proteins and genes being associated with MAMs has been continually on the rise since its discovery. There is an overwhelming overlap between the biochemical functions of MAMs and processes affected in neurodegenerative disorders such as Alzheimer’s disease (AD), Parkinson’s disease (PD), amyotrophic lateral sclerosis (ALS), and Huntington’s disease (HD). Thus, MAMs have received well-deserving and much delayed attention as modulators for ER–mitochondria communication and function. This review briefly discusses the recent progress made in this now fast developing field full of promise for very exciting future therapeutic discoveries.

## 1. Introduction

**Figure 1:**
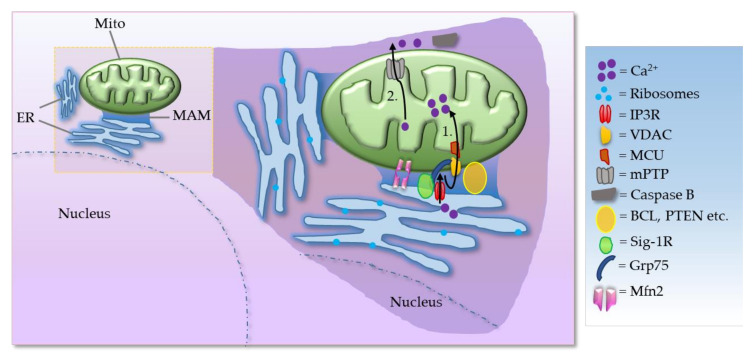
Simplified schematic diagram of basic components of the MAMs. Process 1, 2: Ca^2+^ transfer and apoptosis—shown in black arrows. ER acts as the main source and mitochondria as the sink for Ca^2+^, while MAM has an essential role in Ca^2+^ trafficking. Ca^2+^ is mobilized through IP3R. IP3Rs located at the ER are one of the main Ca^2+^-release channels and upon activation by IP3 can transfer Ca^2+^ to the mitochondria through an IP3R–VDAC–mitochondrial calcium uniporter (MCU) complex. In mammalian cells, IP3R forms a complex with Grp75 and VDAC to maintain ER–mitochondria contact sites. Additionally, mitofusin 2 (Mfn2) also serves as an important tether as described in the text. (Mito = mitochondria; mPTP = mitochondrial permeability transition pore; BCL = B-cell lymphoma 2 family of regulator proteins; PTEN = Phosphatase and tensin homolog).

The physical and functional communication between mitochondria and endoplasmic reticulum (ER) is achieved through close juxtaposition between the two organelles called as mitochondria-associated membranes (MAMs). MAMs are basically ER membranes closely apposed to mitochondria, which can be purified as distinct structures [1]. The tethering of a region of ER to mitochondria is reversible and several specific proteins are transiently localized in the MAM [2]. The tethering of the MAMs acts as a signaling hub for calcium (Ca^2+^) and lipid transfer between ER and mitochondria. The MAMs are also sometimes referred to as the ER–mitochondrial lipid raft-like microdomains since these contain cholesterol and glycosphingolipids and play an important role in the regulation of phospholipid, cholesterol ester, fatty acid metabolism, and lipid droplet formation [3,4]. Cardiolipin is also found at these specialized microdomains at the contact sites of the IMM and OMM. The MAMs also regulate mitochondrial morphology (fusion–division) and motility, mitochondrial bioenergetics, and redox status and play a key role in the modulation of ER stress, proteostasis including autophagy, inflammasome signaling, and apoptosis and cell survival [5]. Despite the fluid nature of the MAMs, these are distinct sub-cellular compartments and there are certain basic components of the MAMs which include 1. Ca^2+^ ion channels located at the ER or at the outer mitochondrial membranes (OMM), e.g., inositol 1,4,5 triphosphate receptor (IP3R), 2. voltage-dependent anion channel (VDAC), 3. enzymes of the lipid biosynthetic pathways and lipid transfer proteins, 4. various chaperones, e.g., glucose-regulated protein 75 (Grp75), calnexin (CNX), sigma 1 receptor (Sig-1R or σ1R), 5. enzymes involved in ER redox regulation, and 6. protein kinases (Figure 1).

**Figure 2:**
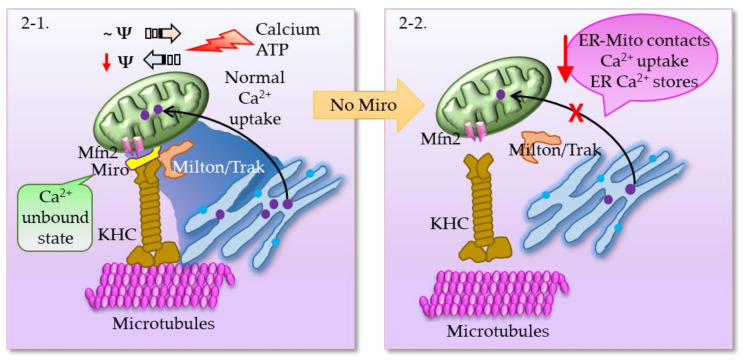
(**2-1**) Mitochondria move along the microtubules in response to changes in the local Ca^2+^ and ATP, in other words, buffering and metabolic demand. Mitochondria with normal membrane potential perform anterograde movement (move towards the periphery), whereas loss of membrane potential results in increased retrograde transport towards the cell body. The cargo adaptor proteins, mitochondrial Rho (Miro) GTPases and Milton, link mitochondria to microtubules via kinesin heavy chain (KHC). Miro binds Milton in a Ca^2+^-unbound state, and thus mitochondria become attached to microtubules. When Miro binds Ca^2+^, it is dislodged from Milton and mitochondria are uncoupled from microtubules. Moreover, Miro1 and Miro2 are required for normal mitochondrial cristae architecture, ER–mitochondria contact sites, and a normal Ca^2+^ uptake. (**2-2**) Absence of Miro results in decreased ER–mitochondria contacts, as a consequence of which ER/mitochondrial handling of Ca^2+^ is severely affected, and there is a significantly larger loss of Ca^2+^ from ER stores.

The VDAC is a key component that mediates Ca^2+^ transport to mitochondria. VDAC interacts physically with IP3R, the ER Ca^2+^ release channel, through the molecular chaperone Grp75. The tripartite complex colocalizes on the MAM and directly enhances the mitochondrial Ca^2+^ uptake. Thus, the VDAC–Grp75–IP3R complex serves as an ER–mitochondria tether [6]. Additionally, deglycase (DJ-1) was recently shown to interact with VDAC, IP3R, and GRP75 [7,8]. Together, IP3R, VDAC, GRP75, and DJ-1 act as a tetramer complex to regulate the transfer of Ca^2+^ from the ER to the mitochondrial matrix via the mitochondrial calcium uniporter (MCU). Grp75 is a member of the heat shock protein 70 family that establishes local contact points between ER and mitochondria. This chaperone protein has a major role in maintaining crosstalk between the two organelles by coordinating the exchange and transfer of Ca^2+^, and in driving subsequent signaling cascades. Grp75 achieves this crucial task through its interaction with both the IP3R and VDAC [6]. Grp75 knockdown and pharmacological inhibition reduced the number of interaction sites between IP3R and VDAC, and thereby reduced ER–mitochondrial coupling [9]. In the MAM, the Sig-1R acts as a molecular chaperone and sustains the proper conformation of the IP3R to ensure flawless Ca^2+^ signaling from the ER into mitochondria to facilitate the production of ATP [10]. Sig-1R binds cholesterol, governs ER lipid compartmentalization, protein trafficking, and Ca^2+^ flux at the MAM [11,12]. At the MAM, the Sig-1R also acts as a chaperone to an ER stress sensor, inositol-requiring enzyme 1 (IRE1), to ensure the proper transmission of ER stress into the nucleus, serving as a conduit between nucleus–ER–mitochondria and resulting in an enhanced production of anti-stress and antioxidant proteins [13]. Moreover, Sig-1R attenuates the formation of reactive oxygen species (ROS) by enhancing the signaling of nuclear factor erythroid 2-related factor 2 (Nrf2) [14]. Mitochondrial Rho (Miro) GTPases, Miro1 and Miro2, are present in the MAMs and regulate normal mitochondrial cristae architecture, mitochondrial division, and the segregation of mitochondria and mitochondrial DNA (mtDNA) in newly generated mitochondrial tips at the ER–mitochondria contact sites [15]. Miro interacts with Mitofusins and the absence of Miro proteins leads to a decrease in contacts between the ER and mitochondria, which correlates with the alterations in mitochondrial Ca^2+^ uptake and in the intraluminal concentration of Ca^2+^ in the ER (Figure 2) [15]. There is evidence for the existence of a regulatory feedback mechanism that can control the number and composition of ER–mitochondrial contacts, depending on the activity of the MAM complexes. Moreover, interaction between Miro and Mfn2 is of interest also because Mfn2 tethers ER to mitochondria [16].

## 2. MAMs in Neurodegenerative Diseases

The cellular homoeostatic functions that are regulated by MAMs are known to be affected in neurodegenerative diseases such as Amyotrophic lateral sclerosis (ALS), Alzheimer’s disease (AD), Huntington’s disease (HD), and Parkinson’s disease (PD). Some of these overlapping abnormalities are briefly discussed below.

### 2.1. MAMs in Amyotrophic Lateral Sclerosis (ALS)

**Figure 3:**
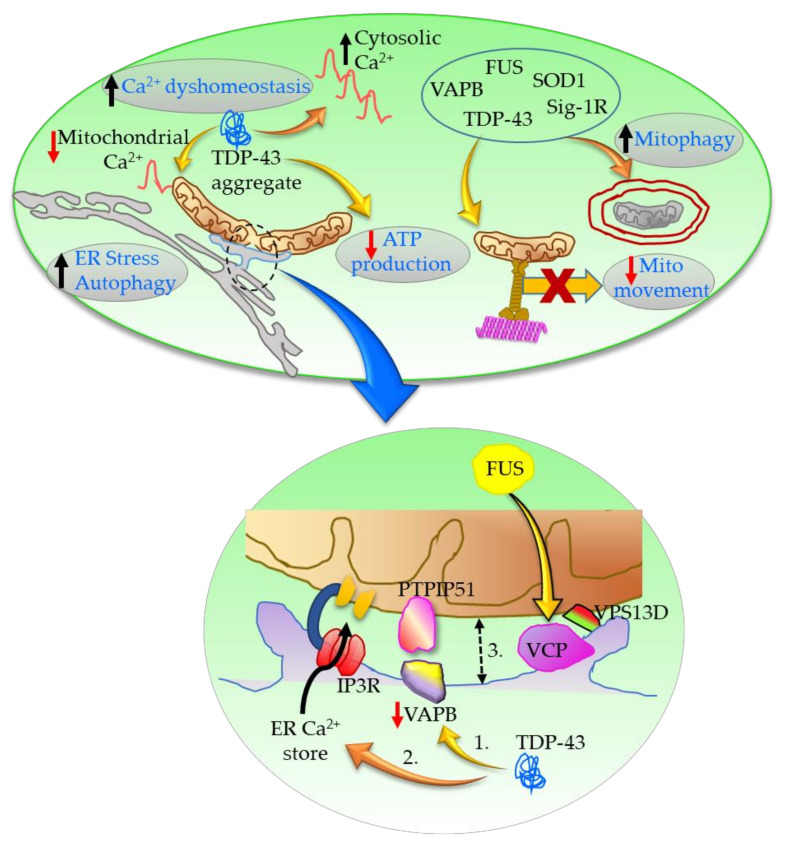
VAPB-PTPIP51 binding regulates ER–mitochondria associations in response to Ca^2+^. In ALS: 1. TDP-43 reduces the amount of VAPB bound to PTPIP51; 2. TDP-43 produces Ca^2+^ dyshomeostasis following IP3R-mediated Ca^2+^ release from ER stores; 3. TDP-43 thus decreases ER–mitochondria associations. Ca^2+^ dyshomeostasis further results in reduced mitochondrial ATP production, impaired anterograde transport of mitochondria, and increased autophagy and ER stress. FUS interacts with VCP and alters VCP–vacuolar protein sorting-associated protein 13D (VPS13D) interactions at the MAM. Furthermore, TDP-43, SOD1, FUS, VAPB, and Sig-1R interact at the MAM to increase mitochondrial fission resulting in mitochondria with decreased membrane potential, and thus increase mitophagy.

ALS is the most common form of motor neuron diseases, for which there is no cure and no effective disease-modifying treatments available. There is progressive muscle paralysis caused by degeneration of upper and lower motor neurons that communicate with the muscle cells. Mutations in the gene encoding for the antioxidant superoxide dismutase 1 (SOD1) and abnormalities found in genes such as TAR-DNA binding protein 43 (TDP43), fused in sarcoma (FUS), ubiquilin 2, vesicle-associated membrane protein B (VAPB), and valosin containing protein (VCP), are all associated with ALS. Among these, VAPB is an integral ER and MAM protein that interacts with protein tyrosine phosphatase-interacting protein-51 (PTPIP51) present in the OMM [17]. VAPB-PTPIP51 binding regulates ER–mitochondria associations while TDP-43 disrupts this binding leading to reduced ER–mitochondria interactions (Figure 3).

TDP-43 increases cytosolic Ca^2+^ and decreases mitochondrial Ca^2+^ levels following IP3R-mediated Ca^2+^ release from ER stores, consistent with a decrease in ER–mitochondria associations [18]. These interruptions in cellular Ca^2+^ homeostasis have far reaching consequences as mitochondrial ATP production, anterograde transport of mitochondria, autophagy, and ER stress are all dependent on it in various ways and are altered in ALS [19,20,21,22,23]. Recently, the VAPB-PTPIP51 were shown to be present in neuronal synapses and to regulate synaptic activity and autophagy [24,25].

Vacuolar protein sorting-associated protein 13D (VPS13D) is a ubiquitously expressed protein that plays an important role in mitochondrial size, autophagy and clearance. VPS13D negatively regulates MAMs partially through its interactions with VCP, an effect that could be reversed by VAPB-PTPIP51 [26]. Functionally, VPS13D suppression leads to severe defects in mitochondrial morphology, mitochondrial cellular distribution, and mitochondrial DNA synthesis. Interestingly, FUS, for which the gene is mutated in ALS, interacts with VCP [27]. These interactions may help explain some of the mitochondrial abnormalities seen in ALS. As mentioned in the ‘Introduction’, Sig-1R is a chaperone that plays important role in the ER–mitochondria Ca^2+^ signaling in MAM and in sensing ER stress. Mutations in Sig-1R were identified in patients with ALS and disruption of Sig-1R in mice led to motor disabilities similar to ALS [28,29]. The loss of function of Sig-1R in the motor neurons disturbed ER–mitochondria contacts, reduced intracellular Ca^2+^ signaling, stunned the axon extension, and was accompanied by activation of ER stress [30]. Disruption of Sig-1R in SOD1 mutant mice accelerated disease onset while also disrupting the integrity of the MAMs. Thus, collapse of the MAM is a common patho-mechanism in both Sig-1R- and SOD1-linked ALS [31]. Furthermore, there is selective enrichment of IP3R3 in motor neurons suggesting that integrity of the MAM is crucial for the selective vulnerability in ALS [31].

### 2.2. MAMs in Alzheimer’s Disease (AD)

AD is the leading cause of dementia among older adults and has a complex etiopathology [32,33]. It is characterized by the accumulation of amyloid β (Aβ) plaques and intracytoplasmic neurofibrillary tangles mostly composed of hyperphosphorylated tau. AD is a progressive disease with advancing age being the most crucial risk factor. There is progressive neuronal loss, particularly in the cerebral cortex and hippocampus, which leads to cognitive impairment. The enzymes β- and γ-secretases cleave amyloid precursor protein (APP), to generate the Aβ peptide. Parallelly, presenilin-1 (PS1) and presenilin-2 (PS2) are enzymatically active components of γ secretase complex contributing to the generation of Aβ peptide. Apolipoprotein E is a regulator of lipid metabolism that has an affinity for Aβ and is another genetic marker that increases the risk of AD. Mutations in APP, PS1, PS2, and apolipoprotein E4 (ApoE4) are associated with an increased risk for and/or as causative factors in AD. Lipid and Ca^2+^ dyshomeostasis, increased levels of circulating cholesterol and mitophagy, impaired mitochondrial dynamics and bioenergetics, and altered glucose metabolism are implicated in AD often preceding appearance of distinct Aβ plaques and tau tangles, as well as before cognitive dysfunction. The disruption of MAM has been implicated in AD pathology, as it is a common denominator in lipid and glucose metabolism, Ca^2+^ homeostasis, ER stress, and mitochondrial function and dynamics [34].

**Figure 4:**
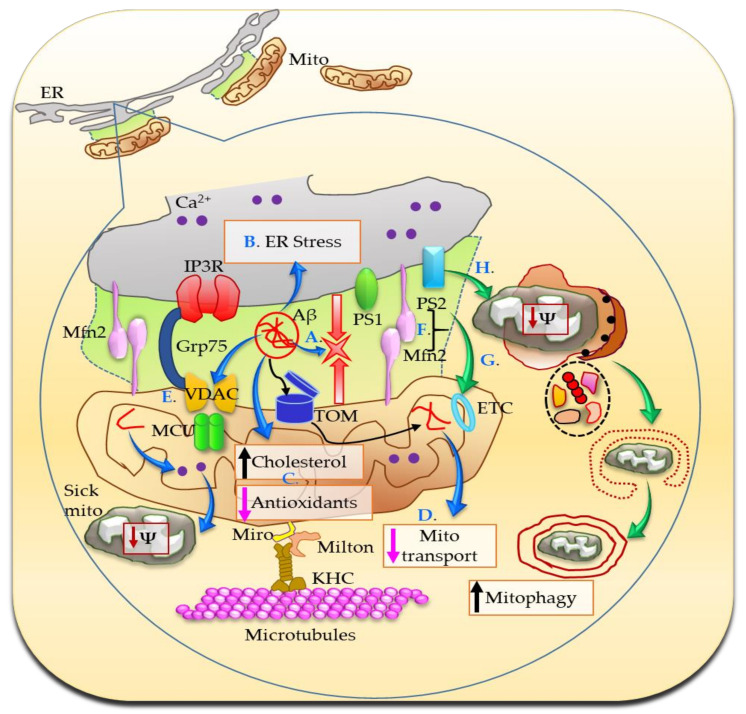
In AD, the MAMs are more abundant and are involved in Aβ production, which further increases ER–mitochondria juxtaposition (A). The translocase of the outer membrane (TOM) situated in the OMM serves as the Aβ transport machinery into the mitochondria. Aβ triggers ER stress (B) and promotes mitochondrial cholesterol accumulation and antioxidant depletion (C). Mutated PS1 and PS2 open IP3R channel flooding intracellular Ca^2+^ and also stimulate Aβ production (not shown). Aβ mediated higher degree of apposition between ER and mitochondria produces exaggerated Ca^2+^ signaling, which further results in abnormal mitochondrial transport (D). Moreover, Aβ, phosphorylated tau, and γ-secretase interact with VDAC resulting in a cascade of toxic effects, including mitochondrial dysfunction and eventually cell death, during the course of AD development and progression (E). Furthermore, PS2 and Mfn2 coordinate to increase ER–mitochondria coupling (F). The excessive Ca^2+^ is toxic for cells especially under stress conditions and over long periods of time, modulates electron transport system (ETC) and increases mitophagy (G,H).

Direct evidence for involvement of the MAM in AD comes from studies demonstrating that cleaved, active forms of the PSs and γ-secretase activity are localized predominantly in the MAM, where PS1 and PS2 interact directly with IP3R, and mutated PS1 and PS2 open IP3R channel flooding intracellular Ca^2+^ and also stimulate Aβ production [35,36,37,38]. The reverse is also true, that is, a genetic reduction of the IP3R by 50%, normalized the exaggerated Ca^2+^ signaling observed in cortical and hippocampal neurons in two mouse models of familial AD. Reduction of IP3R also attenuated Aβ accumulation and tau hyperphosphorylation, and rescued hippocampal long-term potentiation and memory deficit in triple transgenic (3xTg) mice [39]. There is a higher degree of apposition between ER and mitochondria, and MAM-localized functions are significantly increased in cellular and animal models of AD and in cells from patients with AD, which may help explain the higher circulatory levels of cholesterol, lipid droplets, and increased phospholipid synthesis in AD (Figure 4) [40].

In mouse neuroblastoma cell line (N2A) overexpressing the APP familial Swedish mutation (APPswe), Fernandes et al. observed alterations in the MAM composition, ER–mitochondria Ca^2+^ transfer, and decreased mitochondrial dynamics and function [41]. Increased juxtaposition of ER and mitochondria results in increased Ca^2+^ signaling. A direct relationship between altered Ca^2+^ homeostasis at the MAM and the amyloidogenic cascade has been stipulated, where Ca^2+^ directly interacts with and enhances the proteolytic activity of the β-site amyloid precursor protein cleaving enzyme 1 (BACE1; required for the production of the Aβ peptide), exacerbates Aβ formation, and promotes tau hyperphosphorylation [42,43,44]. At the presynaptic terminals, an increased release of Ca^2+^ from the ER stores can undermine synaptic plasticity. Increased Ca^2+^ handling causes the activation of signaling cascades through the modulation of kinases and phosphatases activities, thus affecting synaptic plasticity and cognitive function in AD [42,43,44]. Severe cellular Ca^2+^ overload is highly toxic, causing massive activation of proteases and phospholipases, promoting cell death. Ca^2+^ phosphate precipitates result in swollen mitochondria and damage the cells. Persistent, excessive intracellular Ca^2+^ causes enhanced Ca^2+^ cycling across the mitochondrial membranes, collapse of the proton gradient, and bioenergetic catastrophe [45]. Moreover, Ca^2+^ binding to cyclophilin D positively regulates mitochondrial permeability transition pore (mPTP) opening and in turn cell death. Once opened, mPTP allows the release of apoptotic factors, residing in the intermembrane space, which can trigger apoptosis by both a caspase-dependent and a caspase-independent pathway [46].

Aβ triggers ER stress and promotes cholesterol synthesis and mitochondrial cholesterol accumulation followed by mitochondrial glutathione depletion (Figure 4). ER stress inhibitor 4-phenylbutyric acid prevents these [47]. A proteomics study revealed changes in proteins involved in cholesterol metabolism, in suppressing Aβ accumulation, ER-associated protein degradation (ERAD), oxidative stress response, mitochondrial protein transport, and ATP production. The interaction network analysis revealed a strong relationship between the detected MAM protein changes and AD which preceded the onset of dementia-like symptoms in the APP/PS1 model [48]. Aberrant mitochondrial dynamics, defects in mitochondrial distribution, transport, and morphology could all be attributed to an error in Ca^2+^-mediated mitochondrial function and ER–MAM connection by Aβ (Figure 4) [34,49]. Additionally, VDAC is a hub protein that interacts with more than 150 other proteins, including phosphorylated tau, Aβ, and γ-secretase, contributing to their toxic effects, triggering cell death and potentially leading to the dysfunction of mitochondria during the course of AD development and progression [50,51]. Genetic deletion or knockdown of presenilins alters many autophagy-related proteins resulting in a buildup of autophagosomes and presenilin-deficient cells inefficiently clear long-lived proteins [52]. Mutated PS2 impairs autophagy by causing a block in the degradative flux at the level of the autophagosome-lysosome fusion step. This block is due to PS2′s ability to partially deplete ER Ca^2+^ content, thus reducing cytosolic Ca^2+^ response upon IP3-linked cell stimulations [53]. PS2 and Mfn2 work in tandem to modulate ER–mitochondria coupling. Mfn2 depletion or its binding to PS2 increases tethering between the two organelles as is observed in AD. The strengthened Ca^2+^ crosstalk, under certain conditions such as cellular stress and over long periods of time, may alter bioenergetics functions and/or increase mitochondria-dependent cell death (Figure 4) [54]. The Sig-1R was previously implicated in the pathogenesis of AD: knockdown of Sig-1R caused neurodegeneration and the levels of Sig-1R were found to be reduced in the brain of patients with AD [55]. Thus, there is no ambivalence regarding the important role that MAMs play in AD pathology.

### 2.3. MAMs in Parkinson’s Disease (PD)

PD is the second most prevalent neurodegenerative disorder after AD, afflicting millions of people worldwide. The disease is characterized by loss of dopaminergic neurons of the substantia nigra pars compacta in ventral mid-brain and accumulation of intra-cytoplasmic fibrillary inclusions, called Lewy bodies, mainly composed of the aggregated and misfolded protein α-synuclein (α-syn). Although prevalently cytosolic, α-syn is present in MAMs as shown by Guardia-Laguarta et al. in cell lines and brain tissue from humans and mice [56]. This study further showed that α-syn at MAM modulates mitochondrial morphology downstream of the mitochondrial fusion/fission machinery. PD-related mutations in human α-syn were shown to inhibit this behavior and result in its reduced association with MAM, coincident with a lower degree of apposition of ER with mitochondria, a decrease in MAM function, and an increase in mitochondrial fragmentation [56]. The effect of PD-related mutations in preventing α-syn from exerting its activity at the MAM was attributed to the increased propensity for aggregation of mutant proteins indicating a physical hindrance of MAM functions by mutant α-syn [57].

Mutations in several other genes (PARK2 (Parkin), PARK6 (phosphatase and tensin homolog (PTEN)-induced kinase 1—PINK1), PARK7 (DJ-1), PARK8 (leucine rich repeat kinase 2—LRRK2), PARK9 (ATP13A2)) are implicated as a causal factor in familial PD, although most cases of PD are sporadic. Reduced mitochondrial function and dynamics is a cellular hallmark in PD brains and other PD models containing mutations in α-syn as well as other genes mentioned above. Loss-of-function mutations in DJ-1 (PARK7) are associated with autosomal recessive early onset PD. DJ-1 modulates mitochondrial Ca^2+^ transients induced upon cell stimulation with an IP3 agonist by favoring the ER–mitochondria tethering. On the other hand, a reduction of DJ-1 levels results in mitochondria fragmentation and decreased mitochondrial Ca^2+^ uptake in stimulated cells [58]. Liu et al. showed that DJ-1 is localized to MAM where it physically interacts with and is an essential component of the IP3R-Grp75-VDAC complexes [8]. DJ-1 ablation disrupted the IP3R-Grp75-VDAC complex and reduced ER–mitochondria association. Similar deficits in IP3R-Grp75-VDAC complexes and MAM were found in the brain of DJ-1 knockout mice in vivo. Moreover, DJ-1 levels were reduced in the substantia nigra of sporadic PD patients, which was associated with reduced IP3R-DJ-1 interaction and ER–mitochondria association [8].

#### Interrelationship between Miro, PINK1, Parkin, and Mitophagy

Since the balance of sick vs. healthy mitochondria is a major deciding factor for whether the cell lives or dies, and because the interplay of the factors involved is complex, we thought this section deserves a special mention. Miro is a molecular rheostat that interacts with a broad array of regulatory ER-OMM partners that include PS2-MFN2, B Cell Receptor Associated Protein 31 (BAP31)-fission 1 (Fis1), IP3R-VDAC, VAPB-PTPIP51, and oxysterol-binding protein (OSBP)-related proteins (ORP5/8)-PTPIP51. It helps in regulating Ca^2+^ in the ER lumen through IP3R-VDAC, facilitates metabolism of phospholipids by exchanging lipid transfer proteins via ORP5/8, and is involved in pro-apoptotic pathways via BAP31-Fis1. Parkin is a cytosolic E3 ubiquitin ligase that is mutated in familial forms of PD. The gene encoding PINK1, a serine/threonine kinase, is also mutated in other autosomal recessive cases of PD. PINK1 acts as a molecular sensor of damaged mitochondria (reduced mitochondrial membrane potential/ROS damage) and selectively activates and recruits Parkin to only those mitochondria within a cell that have incurred damage. Together, PINK1 and Parkin act as a mitochondrial surveillance machine to ensure neuronal health. Mutations in PINK1 that are found in PD patients fail to recruit Parkin. Mitophagy, i.e., the selective degradation of mitochondria by the autophagosome, is induced by energetic imbalance or by depolarization of mitochondria. Dissipation of mitochondrial membrane potential impedes the translocation of PINK1 via the translocase of the inner membrane (TIM) channel, resulting in the accumulation of PINK1 in the OMM. Upon damage, PINK1 accumulates on the OMM but is unable to phosphorylate Miro until another PD-related protein, LRRK2, phosphorylates Miro. After Miro is phosphorylated by LRRK2, PINK1 is activated by autophosphorylation and activates the downstream Parkin protein that ubiquitinates several mitochondrial membrane-associated proteins including the ion channel VDAC, the translocase of the outer membrane (TOM) 20, Mfn1, Mfn2, and the Miro protein. Degradation of Miro is an essential step to arrest mitochondrial movement prior to mitophagy. Miro is phosphorylated by PINK1 followed by ubiquitination by Parkin, thus targeting Miro for proteasomal degradation and subsequent mitochondrial clearance via mitophagy.

Effects of Parkin overexpression on ER–mitochondria crosstalk were earlier studied with respect to the regulation of two key cellular parameters: Ca^2+^ homeostasis and ATP production [59]. Parkin overexpression physically and functionally enhanced ER–mitochondria coupling, favored Ca^2+^ transfer from the ER to the mitochondria following stimulation with an IP3 generating agonist, and increased the agonist-induced ATP production. Whereas, Parkin silencing caused mitochondrial fragmentation, impaired mitochondrial Ca^2+^ handling, and reduced the ER–mitochondria tethering [59]. Perturbations in MAM were shown in primary fibroblasts from Parkin knockout mice and PD patients with PARK2 mutations, specifically ER and mitochondria were in closer proximity and Ca^2+^ flux to cytosol and Mfn2 (involved in ER–mitochondria tethering) were increased [60]. Reduction of Mfn2 and increase in Parkin were both able to reverse these alterations. Another independent study observed an increase in contacts between mitochondria and the ER in both flies and cultured human fibroblasts from PD patients with PINK1 or Parkin mutations [61]. In a *Drosophila* model, loss of PINK1 or Parkin led to the activation of ER stress through a direct interaction between mitochondria and the ER, promoted by increased levels of *Drosophila* Mfn. As mentioned previously, Mfn2 forms complexes that tether mitochondria to ER, and Mfn2 is a target for Parkin mediated ubiquitination [16,62]. In this context, it is interesting to note that in Parkin deficient cells and Parkin mutant human fibroblasts, the tether between ER and mitochondria is decreased. Moreover, a non-ubiquitinatable Mfn2 mutant fails to restore ER–mitochondria physical and functional interactions [62]. Furthermore, Mfn2 gates the autophagic turnover of mitochondria by PINK1 and Parkin, and PD-related LRRK2 mutations impair depolarization-induced mitophagy through inhibition of mitochondrial accumulation of LRRK2 substrate indicating that Mfn2, LRRK2, PINK1, and Parkin all converge on a common pathway of mitophagy [63,64,65]. During mitophagy, PINK1-Parkin catalyze a rapid increase in Mfn2 ubiquitination, removing Mfn2 tether to dislodge mitochondria from ER; MAMs tear apart, and the reduction in ER–mitochondria appositions increases the rate of mitochondrial degradation. Additionally, it was shown that following mitophagic stimuli, PINK1 interacts with the proautophagic protein Beclin1 and colocalizes with it at the MAM [66]. Together Beclin 1 and PINK1 promote the enhancement of ER–mitochondria contact sites and the formation of omegasomes that represent autophagosome precursors. This study also highlighted that Parkin is also enhanced at the MAM following mitophagy induction and that PINK1 acts upstream of Parkin [66]. Recently, it was stipulated that LRRK2 plays a role in ER–mitochondrial tethering where it regulates the activities of E3 ubiquitin ligase Parkin among others via kinase-dependent protein–protein interactions [67]. It is relevant to mention here that mitophagy is compromised in PD patients and various models of PD, which may be attributed to the above mentioned complex interactions between PINK1, Parkin, Mfn2, LRRK2, and other factors at the MAM [68].

Another potential point of interference of MAM in PD is the dopamine receptors (DR) and Sig-1R interactions. DRs play crucial roles in many neurological processes, including motivation, cognition, memory, and motor function. Heterodimerization of Sig-1R and dopamine D1 receptor (D1R) and D2R has been reported in vitro and in vivo [69]. In a mouse model of experimental Parkinsonism (intrastriatal 6-hydroxydopamine lesions) gradual and significant improvement of motor performance was observed following treatment with a selective Sig-1R agonist [70]. The behavioral recovery was paralleled by an increased density of dopaminergic fibers in the most denervated striatal regions, by a modest recovery of dopamine levels, and by an upregulation of neurotrophic factors (brain-derived neurotrophic factor (BDNF) and glial cell line-derived neurotrophic factor (GDNF)) and their downstream effector pathways (extracellular signal regulated kinases 1/2 and protein kinase B (Akt)). Further, this agonist treatment also caused a wider intracellular distribution of Sig-1Rs, presumably due to the agonist-induced Sig-1R translocation. As expected, these beneficial effects were ablated in Sig-1R knockout mice [70]. These results suggest that Sig-1R regulates endogenous defense and plasticity mechanisms.

### 2.4. MAMs in Huntington’s Disease (HD)

HD is an incurable autosomal-dominant progressive neurodegenerative disease for which the cause is known for years and it is an expansion of the cytosine–adenine–guanine (CAG) repeats in the coding sequence of the mutant huntingtin gene. The product, mutant huntingtin protein, is prone to proteolytic cleavage, misfolding, and aggregation. HD is characterized by selective degeneration of gamma-aminobutyric acid (GABA)-ergic medium spiny neurons in the striatum, although other brain regions are known to be affected later in the course of the disease. In HD, mitochondrial dysfunction and energy impairments occur before overt pathological symptoms and appear to be central in driving the progression of the disease. At the same time, mitochondrial fission/fusion alterations, including Mfn1, Mfn2, optic atrophy 1 (Opa1), dynamin-related protein 1 (Drp1), mitochondrial movements, and mitochondrial Ca^2+^ handling are affected in HD and have been well investigated and described [71,72,73,74,75,76,77,78]. It was shown that mutant huntingtin binds to IP3R and causes sensitization of IP3R to activation by IP3 in planar lipid bilayers and in primary medium spiny neurons, the neuronal population affected foremost in HD. Moreover, a connection between abnormal Ca^2+^ signaling and apoptosis of medium spiny neurons was shown in primary culture from a YAC128 HD mouse model expressing the full-length mutant human huntingtin gene [79]. The medium spiny neurons are extremely sensitive to changes in the cytoplasmic concentration of Ca^2+^ and its excessive increase leads to their death. Recently, targeting Sig-1R through a drug based on its agonists has been suggested as an approach to normalize the balance of Ca^2+^ in striatal neurons [80]. Pridopidine is one such selective Sig-1R agonist, currently in clinical development for HD and ALS. Pridopidine has the capacity to be a dopamine buffer, although it has been reported that pridopidine has a 100-fold greater affinity for the Sig-1R than for the D2 receptor. Pridopidine prevents the disruption of mitochondria–ER contact sites and improves the co-localization of IP3R and Sig-1R in primary neurons from YAC128 HD mice, leading to increased mitochondrial activity, elongation, and motility [81]. These effects were associated with an increase in mitochondrial respiration in pridopidine-treated YAC128 HD neurons and human HD neural stem cells. YAC128 neurons, human HD neural stem cells, and human HD lymphoblasts show increased ROS levels and deficient antioxidant response, which are efficiently rescued with pridopidine. Moreover, YAC128 HD mice treated at early/pre-symptomatic age with pridopidine showed significant improvement in motor coordination [81]. These studies show that pridopidine treatment results in neuroprotective effect, which is manifested as an increase in the plasticity of synaptic neurons and prevention of their atrophy within the striatum and provide proof-of-concept for MAM targeted therapies in HD [82,83,84,85]. Interestingly, Sig-1R itself accumulates in neuronal inclusions containing mutant huntingtin where it likely increases proteasome activity and mutant huntingtin degradation [86].

Since HD pathology has multiple intersections with the impairment in peroxisome proliferator-activated receptor (PPAR)-γ coactivator-1α (PGC-1α), a transcriptional master co-regulator of mitochondrial biogenesis, metabolism, and antioxidant defenses, it is tempting to speculate that PGC-1α at the ER–mitochondria interface has an important role to play, just waiting to be explored. PGC-1α ablation in mice shows HD-like pathology in brown adipose tissue and striatum as well as motor abnormalities similar to HD [87,88]. In HD, PGC-1α levels and activity are reduced in the brain and peripheral tissues from patients and transgenic models [89,90,91]. Parkin controls the expression of PGC-1α and PGC-1α target gene nuclear respiratory factor-1 (NRF-1), via Parkin interacting substrate (PARIS) by binding to insulin response sequences in the PGC-1α promoter [92]. Parkin and PGC-1α functionally interact to regulate the turnover and quality of mitochondria, by increasing both mitophagic activity and mitochondrial biogenesis, exceeding the effects seen with PGC-1α alone [93]. Co-expression of PGC-1α and Parkin increases the number of mitochondria and maximal respiration and accelerates the recovery of the mitochondrial membrane potential following mitochondrial uncoupling in cortical neurons. Moreover, PGC-1α increases the turnover of Mfn2 by increasing both its transcription as well as degradation via ubiquitylation by Parkin on mitochondria. Of particular interest are the studies showing Parkin and PGC-1α together have neuroprotective effects and control the density of mitochondria, and also control the interaction of mitochondria with the ER [93].

Chronic ER stress is a side effect of mutant huntingtin since it inhibits ER-associated degradation, ER/Golgi vesicular trafficking and axonal transport, and disrupts autophagy and ER Ca^2+^ homeostasis resulting in disturbance of protein folding and maturation pathways at the ER [94,95,96]. Reducing misfolded protein accumulation in the ER reduces ER stress and is protective in HD. As another point of intersection between ALS and HD, VCP is sequestered by mutant huntingtin and its overexpression resolved ER-associated degradation and ER stress [97]. Since mutant huntingtin aggregates directly interact with both mitochondria and ER, it is pertinent to assume that mutant huntingtin functionally and physically interferes with factors at MAM. It was demonstrated in several models of HD that VCP selectively translocates to mitochondria where it binds mutant huntingtin, causing excessive mitophagy and neuronal death [98]. Modulating VCP-mutant huntingtin interaction with small molecule therapeutics has protective effects in HD mouse- and patient-derived cells and HD transgenic mouse brains [98,99,100].

## 3. Conclusions and Future Directions

**Figure 5:**
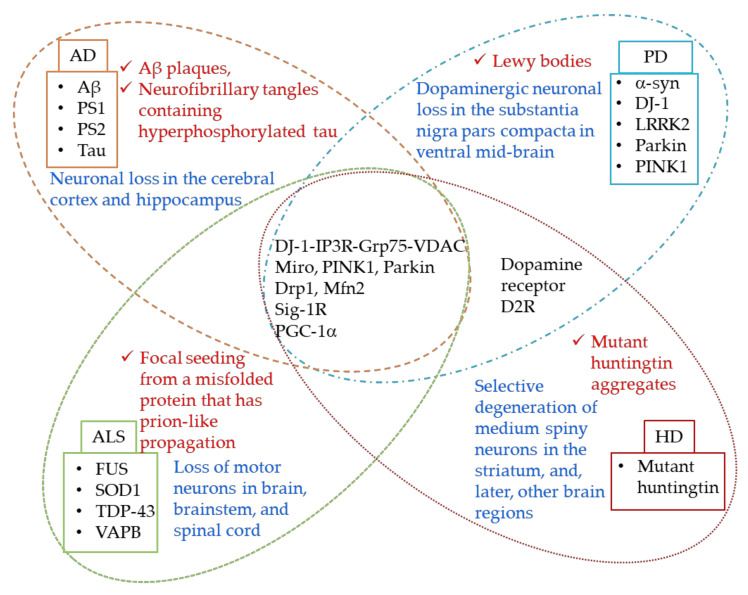
Although certain factors are different, there are significant overlaps between AD, PD, HD, and ALS when it comes to ER–MAM–mitochondria interactions. Apart from obvious endgame similarities i.e., Ca^2+^ dyshomeostasis, ETC complex disturbances, increased fission/fusion, mitophagy, antioxidant depletion, ROS, ER stress and apoptosis, and reduced mitochondrial movement, mechanistic commonalities include alterations in the following: 1. DJ-1-IP3R-Grp75-VDAC tetrameric complex. DJ-1 interacts with IP3R-Grp75-VDAC and is an essential part of the complex. The release of Ca^2+^ from ER is mediated via this complex; 2. Sig-1R, IP3R, D2R interactions. Sig-1R interacts with Dopamine receptors and regulates plasticity mechanisms; 3. Miro, PINK1, Parkin, and Mfn2 together regulate mitophagy and ER–mitochondria connections; 4. PGC-1α is altered in all these diseases and interacts with both Parkin and Mfn2, thus it is logical to speculate a role of this important cofactor at the MAMs. Proteins mutated in each of the diseases are shown in their respective boxes. Neuropathological hallmarks of each disease are given in red, while the selective neuronal population affected is shown in blue.

The MAMs serve as important crossover points for the information relay between ER and mitochondria, and regulate many functions of mitochondria, including maintaining Ca^2+^ balance, mitochondria shape, size and dynamics, and play an indispensable role in cell survival. There is vast literature on these functions being affected in neurodegeneration and mysteries are just beginning to unravel. Despite the apparent dissimilarities in clinical presentations of AD, PD, HD, and ALS, the MAM dysfunction appears to be a common denominator at the mechanistic level. Moreover, there are structural and functional factors involved at the MAM that are commonly perturbed in all the neurodegenerative diseases that are discussed in this review (Figure 5).

Persistent alterations in Ca^2+^ flux to cytosol, and in the proteins involved in ER–mitochondria tethering, e.g., Mfn2, Sig-1R, etc., are detrimental to the cell [101]. At the same time, direct targeting of these factors at the MAM using agonists or antagonists provides therapeutic opportunities for these devastating illnesses [102]. For example, manipulation of ER–mitochondria contacts using an ER–mitochondria synthetic linker rescues the locomotor deficit in a *Drosophila* model of PD [62]. Since ER–mitochondria associations at the MAM (lipid raft-like microdomains) are central in correct folding as well as distribution of mature proteins to the appropriate locations within the cell, it is tempting to speculate that the misfolding and aggregation of the various mutated proteins (e.g., mutant huntingtin in HD, α-synuclein in PD, etc.) starts at the MAM. Targeting MAM thus presents a viable and attractive therapeutic opportunity.

Correcting mitochondrial bioenergetics, biogenesis, and dynamics (fission, fusion, motility) through PGC-1α mediated pathways is another potential therapeutic avenue. PGC-1α is altered in all four diseases, and PGC-1α ablation is detrimental to both, the ER as well as the mitochondria [87,88,103,104]. In addition to the well described mitochondrial abnormalities, PGC-1α deletion in mice results in altered ER morphology, and fragmented cisternae with disrupted stack arrangement. Additionally, PGC-1α alters the MAM contacts between the ER and the mitochondria [103]. PGC-1α, together with Parkin, controls Mfn1, Mfn2, and VDAC, as well as the density, quality, and turnover of mitochondria and their interaction with the ER [93]. These, in turn, affect behavior, cognition, and neuronal survival. There are various ways to modulate PGC-1α function including, diet, exercise, caloric restriction, and small molecule mediated therapies [91,105,106,107]. Another approach to enhancing cell survival is to reduce Parkin-mediated excessive mitophagy by reducing the ubiquitination of mitochondrial outer membrane proteins Mfn, VDAC, Fis1, and TOM20. Progress has been made to identify such de-ubiquitinating enzymes (DUBs) that oppose Parkin in the ubiquitination of its targets [108]. Such enzymes may be crucial in developing specific isopeptidase modulators that will help maintain healthy mitochondria and prevent overactive mitophagy.

The open question, however, remains whether MAM dysfunction is causally linked to the disease pathogenesis in AD, PD, HD, and ALS. Another critical turning point is the dynamic nature of the MAMs owing to which detailed molecular maps need to be further characterized in the context of various cell types. This will help in identifying putative drug candidates for disease-specific treatments. Nevertheless, in view of the importance of the MAMs for essential functions and survival of the neurons, along with the initial findings of neuroprotection imparted by targeting the MAMs, it is only a matter of time before the light at the end of the tunnel becomes a reality in terms of an effective therapy for some of these incurable diseases.
